# Epidemiology and socioeconomic correlates of brain and central nervous system cancers in Asia in 2020 and their projection to 2040

**DOI:** 10.1038/s41598-024-73277-z

**Published:** 2024-09-20

**Authors:** Seyed Ehsan Mousavi, Homa Seyedmirzaei, Shahrzad Shahrokhi Nejad, Seyed Aria Nejadghaderi

**Affiliations:** 1 Neurosciences Research Center, Aging Research Institute, Tabriz University of Medical Sciences, Tabriz, Iran; 2 Department of Community Medicine, Social Determinants of Health Research Center, Faculty of Medicine, Tabriz University of Medical Sciences, Tabriz, Iran; 3 Sports Medicine Research Center, Neuroscience Institute, Tehran University of Medical Sciences, Tehran, Iran; 4 Interdisciplinary Neuroscience Research Program (INRP), Tehran University of Medical Sciences, Tehran, Iran; 5 School of Medicine, Shahid Beheshti University of Medical Sciences, Tehran, Iran; 6 HIV/STI Surveillance Research Center, and WHO Collaborating Center for HIV Surveillance, Institute for Futures Studies in Health, Kerman University of Medical Sciences, Kerman, Iran; 7 Systematic Review and Meta‑analysis Expert Group (SRMEG), Universal Scientific Education and Research Network (USERN), Tehran, Iran

**Keywords:** Brain neoplasm, Central nervous system neoplasm, Meningioma, Epidemiology, Incidence, Mortality, GLOBOCAN, Cancer epidemiology, CNS cancer

## Abstract

Brain and central nervous system (CNS) cancers constitute a heterogeneous group of cancers with poor 5-year survival rates. We aimed to report the epidemiology of brain and CNS cancers in Asia in 2020 and their projections up to 2040 by age, sex, and country, as well as their correlation with socioeconomic status. We extracted data from the 2020 Global Cancer Observatory (GLOBOCAN). Numbers, age-standardized incidence rates (ASIRs) and mortality rates (ASMRs), 5-year prevalent cases and rates, mortality-to-incidence ratios (MIRs), and crude rates were calculated. The human development index (HDI) and current healthcare expenditure (CHE)-to-gross domestic product (GDP) ratio were included as indicators of socioeconomic status. Additionally, the numbers of new cases and deaths were predicted from 2025 to 2040 by multiplying the anticipated population during this period by age-standardized rates. In 2020, there were 166,925 new cases of brain and CNS cancers in Asia, indicating a 5-year prevalence rate of 9.40 per 100,000. We also estimated the total ASIR, ASMR, and MIR as 3.20, 2.60, and 0.83, respectively. There were significant negative correlations between HDI and MIR (correlation coefficient: − 0.538, p value < 0.001) and significant positive correlations between CHE/GDP% and ASIR (correlation coefficient: 0.388, p value: 0.010) and ASMR (correlation coefficient: 0.373, p value: 0.014). In 2040, there will be 232,000 new cases of brain and CNS cancers and 200,000 subsequent deaths in Asia. Our study revealed higher brain and CNS cancer rates in Western Asia among males and elderly individuals. These findings can aid policymakers in enhancing cancer care and suggest the consideration of risk factors in future research.

**Supplementary Information:**

The online version contains supplementary material available at 10.1038/s41598-024-73277-z.

##  Introduction

Brain and central nervous system (CNS) cancers are a group of heterogeneous and complex cancers that originate from the brain and adjacent tissues and have a 5-year survival rate of approximately 36%, with a poor prognosis for both glioblastoma patients and the elderly^1–3^. Glioblastoma is the most common type of CNS cancer in Asia^4^. Glioblastoma was more common in males, whereas meningioma was more common in females. Additionally, in children under 14 years of age, both malignant and nonmalignant CNS cancers are the most prevalent type of solid cancers^4,5^. Male sex and higher socioeconomic status are associated with brain cancer burden^6^. However, risk factors, with genetics and ionizing radiation exposure as leading etiological factors, are rather unknown^1,6^. Depending on the affected site, symptoms, such as seizures, headache, and elevated intracranial pressure, can be focal or generalized^7^. Owing to recent developments in imaging techniques, changes in exposure to environmental risk factors, and extended lifespan, more cases of brain and CNS cancers are predicted to be diagnosed worldwide^2^. Therefore, regular updates on the epidemiology and changes in the trends of these cancers are necessary^2^.

In 2020, brain and CNS cancers ranked 21st in terms of incidence rates among 36 types of cancers worldwide^8^. In 2019, the global age-standardized mortality rate (ASMR) and age-standardized incidence rate (ASIR) of brain and CNS cancers were 3.05 and 4.34 per 100,000, respectively^2,9^.

Reporting the epidemiology of cancer in Asia is highly important since 59.5% of the global population resides on this continent, and these findings can be helpful for regional health policymaking^8^. Moreover, 49.3% of all new cases of cancer and 58.3% of all cancer deaths in 2020 occurred in Asia^8^. From 2010 to 2019, brain and CNS cancers ranked 10th in terms of disability-adjusted life years^9^. As the highest proportion of patients in the world are in China, the United States, and India, the significant contribution of Asia to the burden of brain and CNS cancers worldwide cannot be ignored^4^. The projections of brain and CNS cancers in Asia from 2020 to 2044 show an increase in ASIRs, whereas the ASMRs gradually decrease^4^.

Previous studies have provided epidemiological data on the incidence, mortality, and changes in the trends of brain and CNS cancers across Asia and worldwide up to 2019^2,4^. The article by Liu and colleagues used the Global Burden of Disease (GBD) 2019 study, provided the estimated annual percent changes, and predicted the burden up to 2044. The study has several limitations: (1) they used data up to 2019, while more recent updated data are necessary for health policy making, (2) they did not report prevalence rates, and (3) relevant statistics and measures about the association between cancer care and socioeconomic development like ratio of mortality-to-incidence or associations with human development index (HDI) was not considered^4^. Additionally, they have reported the epidemiology by income level^10^ or in some specific countries^11,12^. We aimed to report the 5-year prevalence, incidence, and mortality of brain and CNS cancers in Asia for each age group and Asian subregion, by sex, and for each country in 2020. Moreover, the correlation between the metrics and the socioeconomic levels, in terms of the HDI and the current healthcare expenditure to gross domestic product ratio (CHE/GDP%), was investigated. We also forecasted the projection of incidence and mortality up to 2040.

## Methods

### Data sources

We gathered epidemiological figures of brain and CNS cancers with the International Classification of Disease codes C70-72 from the GLOBOCAN database. The World Health Organization (WHO) and the International Agency for Research on Cancer established this public access database, which details epidemiological figures for 36 main types of cancer standardized for age and sex in 30 regions of the world and 185 countries^13^. The methods used in gathering data from sources have been described in previous studies^8^. We extracted the incidence and mortality rates standardized by age, sex, and country. The nations’ 2020 incidence and mortality rates are projected for the population in 2020. Countries’ registries for cancer provided national and adjacent countries’ incidence and mortality rates, and the rates were calculated with the model of mortality-to-incidence ratios (MIRs).

### Study variables

The incidence and mortality rates of brain and CNS cancers were collected, followed by the calculation of MIRs by dividing the crude mortality rate by the incidence rate. MIR, which represents the quality of care in the healthcare setting, has lower values when screening and overall cancer management care are superior to other equally derived MIRs in our study^14^. In addition, to report the progression and mortality risk of brain and CNS cancers in individuals under the age of 75 years, we noted the incidence and mortality cumulative risk percentages. A further indicator in our study was CHE/GDP%, which represents the financial allocation to healthcare in a country and signifies the pivotal contribution of healthcare to the total economy.

The HDI was incorporated to consider the socioeconomic level of a country. It is a universally used composite index of life expectancy, mean education acquired by 25-year-old and older individuals, the predicted duration of school-aged children’s education, and per capita income indicators^15^.

Briefly, the data in our study were categorized by age, sex, region (North America, Latin America and the Caribbean, Europe, Africa, and Oceania) Asian subregion (Western [Turkey, Iraq, Israel, Azerbaijan, Syrian Arab Republic, Yemen, Saudi Arabia, Georgia, Jordan, Armenia, Lebanon, Gaza Strip and West Bank, United Arab Emirates, Oman, Bahrain, Kuwait, and Qatar], South Central [India, Iran, Pakistan, Uzbekistan, Bangladesh, Kazakhstan, Afghanistan, Sri Lanka, Kyrgyzstan, Turkmenistan, Tajikistan, Nepal, and Bhutan], Eastern [China, Japan, Republic of Korea, Democratic Republic of Korea, and Mongolia], and Southeastern Asia [Indonesia, Viet Nam, Thailand, Philippines, Myanmar, Malaysia, Cambodia, Singapore, Lao People’s Democratic Republic, Brunei Darussalam, and Timor-Leste]), and Asian country.

### Statistical analysis

The average total rates from adjacent countries were used to calculate all national age- and sex-adjusted incidence rates of cancer. We then calculated the 5-year incidence of brain and CNS cancers by using the ratio of incidence to five-year incidence (adjusted by age, sex, and country) of the Nordic regions from 2006 to 2015, as illustrated below:

$$\:{Prevalence}_{Country}=\:{Incidence}_{Country}\times\:\:\frac{{Prevalence}_{Nordic}}{{Incidence}_{Nordic}}\:\times\:\:\frac{{HDI}_{Country}}{{HDI}_{Nordic}}$$

To estimate the rates, we extracted the 2020 population data from the World Population Prospects of the United Nations, which were projected and last revised in 2019. Additionally, we extracted HDI data from the Human Development Report Office of the United Nations Development Programme^15^. The Global Health Observatory data repository of the WHO was used to extract data on CHE as a proportion of GDP in 2019^16^. The design of our study and the results are in accordance with the Guidelines for Accurate and Transparent Health Estimates Reporting^17^ and the Strengthening the Reporting of Observational Studies in Epidemiology statements^18^.

The figures and tables in our study provide the incidence and mortality cases, crude incidence and mortality rates, prevalent cases and rates of 5-year prevalence, ASMRs, and ASIRs. All rates were considered to be expressed in 100,000 people. Standardization was performed directly according to the 1966 Segi-Doll World standard population. Age groups were stratified with intervals of 10 or five years. Furthermore, the bivariate correlation test analyzed the associations between ASIRs, ASMRs, and MIRs of brain and CNS cancers with the CHE/GDP% and HDI of the countries with existing data. Pearson’s correlation coefficient was employed, and the results were stratified into three categories: weak (< 0.3), moderate (0.3–0.5), and strong (> 0.5) correlations. A statistically significant p value was considered < 0.05 in a two-sided test. The number of new cases and deaths from 2025 to 2040 were predicted through the multiplication of the anticipated population by the 2020 ASIRs and ASMRs. The 95% uncertainty intervals (UIs) for the estimated number of new cancer cases and cancer deaths, broken down by sex and site for all ages, were calculated using the standard error of the crude incidence or mortality rates used in the estimation. The cumulative risk percentages were calculated by determining the likelihood that an individual would develop or die from brain and CNS cancers before the age of 75. This was done using the age-specific incidence and mortality rates collected for each population group. The cumulative risk was computed by summing the age-specific rates across all age intervals up to 75 years and then applying standard life table methods to estimate the cumulative probability of cancer incidence or mortality within that age range. Version 4.3.2 of R statistical software was used for statistical analysis^19^.

## Results

### Prevalence, incidence, mortality, and MIR in the globe and Asia in both sexes

Worldwide, there were 837,152 cases of brain and CNS cancers diagnosed within the previous five years in 2020, with a 5-year prevalence rate of 10.70 per 100,000. Moreover, there were 308,102 estimated new cases of brain and CNS cancers in 2020 (95% UI: 295,692.0–321,033.0), indicating a crude rate of 4.00 per 100,000, an ASIR value of 3.50 per 100,000, and a cumulative risk of 0.61%. In 2020, brain and CNS cancers led to 251,329 estimated deaths worldwide (95% UI: 244,434.0–258,418.0), representing a crude rate of 3.20 per 100,000, an ASMR of 2.80 per 100,000, and a cumulative risk of 0.54%. Accordingly, we measured the MIR of brain and CNS cancers equal to 0.80 globally (Table 1).

**Table 1:** Five-year prevalence, incidence, and mortality metrics in 2020 of brain and central nervous system cancers for different geographic location in both sexes, males, and females.

Location	Prevalence		Incidence					Mortality					MIR
	5-year prevalent cases	5-year prevalence rate	Number	Uncertainty interval	Crude rate	ASIR	Cumulative risk (%)	Number	Uncertainty interval	Crude rate	ASMR	Cumulative risk (%)	
Both sexes													
World	837,152	10.70	308,102	295692.00–321033.00	4.00	3.50	0.61	251,329	244434.00–258418.00	3.20	2.80	0.54	0.80
Asia	435,532	9.40	166,925	159699.00–174478.00	3.60	3.20	0.52	137,646	131171.00–144440.00	3.00	2.60	0.47	0.83
Eastern Asia	242,242	14.40	89,131	86514.80–91826.40	5.30	3.80	0.61	71,504	70563.10–72457.50	4.30	2.90	0.52	0.81
South-Central Asia	116,123	5.80	48,322	45034.80–51849.10	2.4	2.50	0.39	41,043	38273.50–44013.00	2.00	2.10	0.36	0.83
South-Eastern Asia	42,852	6.40	16,643	15218.70–18200.60	2.50	2.40	0.38	14,361	13086.10–15760.10	2.10	2.00	0.36	0.84
Western Asia	34,315	12.30	12,829	11551.10–14248.20	4.60	5.00	0.96	10,738	10130.40–11382.00	3.90	4.20	0.89	0.85
Continents													
Africa	41,311	3.10	18,264	14369.90–23213.30	1.40	1.90	0.35	15,157	11539.00–19909.30	1.10	1.70	0.34	0.79
Europe	197,846	26.40	67,114	64949.40–69350.70	9.00	5.70	0.91	53,680	52043.40–55368.10	7.20	4.00	0.76	0.80
Latin America and the Caribbean	68,999	10.60	25,835	23945.60–27873.40	4.00	3.50	0.64	22,176	21483.30–22891.10	3.40	2.90	0.60	0.85
Northern America	85,937	23.30	27,526	27169.30–27887.40	7.50	5.40	0.83	20,690	20343.20–21042.80	5.60	3.30	0.68	0.75
Oceania	7527	17.60	2438	2292.30–2593.00	5.70	4.30	0.78	1980	1846.50–2123.20	4.60	3.30	0.70	0.81
Males													
World	425,258	10.80	168,346	159163.00–178059.00	4.30	3.90	0.70	138,277	129473.00–147680.00	3.50	3.20	0.63	0.81
Asia	222,787	9.40	91,992	86579.50–97742.80	3.90	3.50	0.59	76,169	71192.70–81493.10	3.20	2.90	0.53	0.82
Eastern Asia	115,555	13.50	46,205	44327.20–48162.40	5.40	4.00	0.66	37,129	35178.70–39187.40	4.30	3.10	0.57	0.79
South-Central Asia	66,904	6.40	29,452	26907.20–32237.40	2.80	3.00	0.49	24,978	22831.80–27325.90	2.40	2.60	0.45	0.85
South-Eastern Asia	22,393	6.70	9211	8145.00–10416.50	2.80	2.70	0.45	8037	7072.90–9132.50	2.40	2.40	0.43	0.85
Western Asia	17,935	12.30	7124	6198.20–8188.00	4.90	5.50	1.05	6025	5566.20–6521.60	4.10	4.70	0.98	0.83
Continents													
Africa	20,761	3.10	9666	6989.50–13367.40	1.40	2.10	0.42	8058	5572.90–11651.20	1.20	1.90	0.43	0.85
Europe	98,825	27.30	36,192	34575.40–37884.20	10.00	6.70	1.09	29,373	28148.10–30651.20	8.10	4.80	0.93	0.81
Latin America and the Caribbean	33,943	10.60	13,581	12221.80–15091.30	4.20	4.00	0.71	11,756	11254.60–12279.80	3.70	3.40	0.68	0.88
Northern America	44,860	24.60	15,483	15215.80–15754.90	8.50	6.30	0.99	11,721	11459.80–11988.10	6.40	3.90	0.83	0.75
Oceania	4082	19.10	1432	1321.00–1552.30	6.70	5.20	0.96	1200	1097.20–1312.50	5.60	4.10	0.88	0.83
Females													
World	411,894	10.70	139,756	131560.00–148462.00	3.60	3.00	0.53	113,052	109663.00–116546.00	2.90	2.40	0.47	0.81
Asia	212,745	9.40	74,933	70236.40–79943.60	3.30	2.80	0.46	61,477	57294.00–65965.40	2.70	2.30	0.41	0.82
Eastern Asia	126,687	15.40	42,926	41127.20–44803.40	5.20	3.60	0.57	34,375	33945.90–34809.40	4.20	2.70	0.48	0.81
South-Central Asia	49,219	5.00	18,870	16865.20–21113.00	1.90	1.90	0.30	16,065	14375.40–17953.20	1.60	1.70	0.27	0.84
South-Eastern Asia	20,459	6.10	7432	6523.60–8466.90	2.20	2.00	0.33	6324	5522.70–7241.60	1.90	1.70	0.31	0.86
Western Asia	16,380	12.40	5705	4855.10–6703.70	4.30	4.40	0.87	4713	4325.00–5135.80	3.60	3.60	0.81	0.84
Continents													
Africa	20,550	3.10	8598	6020.90–12278.20	1.30	1.70	0.29	7099	4733.80–10645.90	1.10	1.50	0.29	0.85
Europe	99,021	25.60	30,922	29500.50–32412.00	8.00	4.80	0.77	24,307	23235.50–25427.90	6.30	3.20	0.62	0.79
Latin America and the Caribbean	35,056	10.50	12,254	10983.50–13671.50	3.70	3.20	0.58	10,420	9948.60–10913.70	3.10	2.60	0.54	0.84
Northern America	41,077	22.10	12,043	11805.70–12285.10	6.50	4.70	0.69	8969	8740.30–9203.70	4.80	2.70	0.56	0.74
Oceania	3445	16.20	1006	914.30–1106.90	4.70	3.50	0.62	780	697.70–872.00	3.70	2.50	0.53	0.79

*ASIR* age-standardized incidence rate, *ASMR* age-standardized mortality rate, *MIR* mortality-to-incidence ratio. Rates are presented per 100,000 population.

In Asia, the estimated 5-year prevalence rate of brain and CNS cancers was 9.40 per 100,000, indicating a total of 435,532 cases. Additionally, 166,925 new cases were estimated of brain and CNS cancers (95% UI: 159,699.0–174,478.0), representing a crude rate of 3.60 per 100,000, an ASIR value of 3.20 per 100,000, and a cumulative risk of 0.52%. These cancers led to 137,646 estimated deaths in Asia in 2020 (95% UI: 131,171.0–144,440.0), resulting in a crude rate of 3.00 per 100,000, an ASMR of 2.60 per 100,000, and a cumulative risk of 0.47%. The MIR of these cancers was 0.83 in Asia (Table 1).

**Fig. 1:**
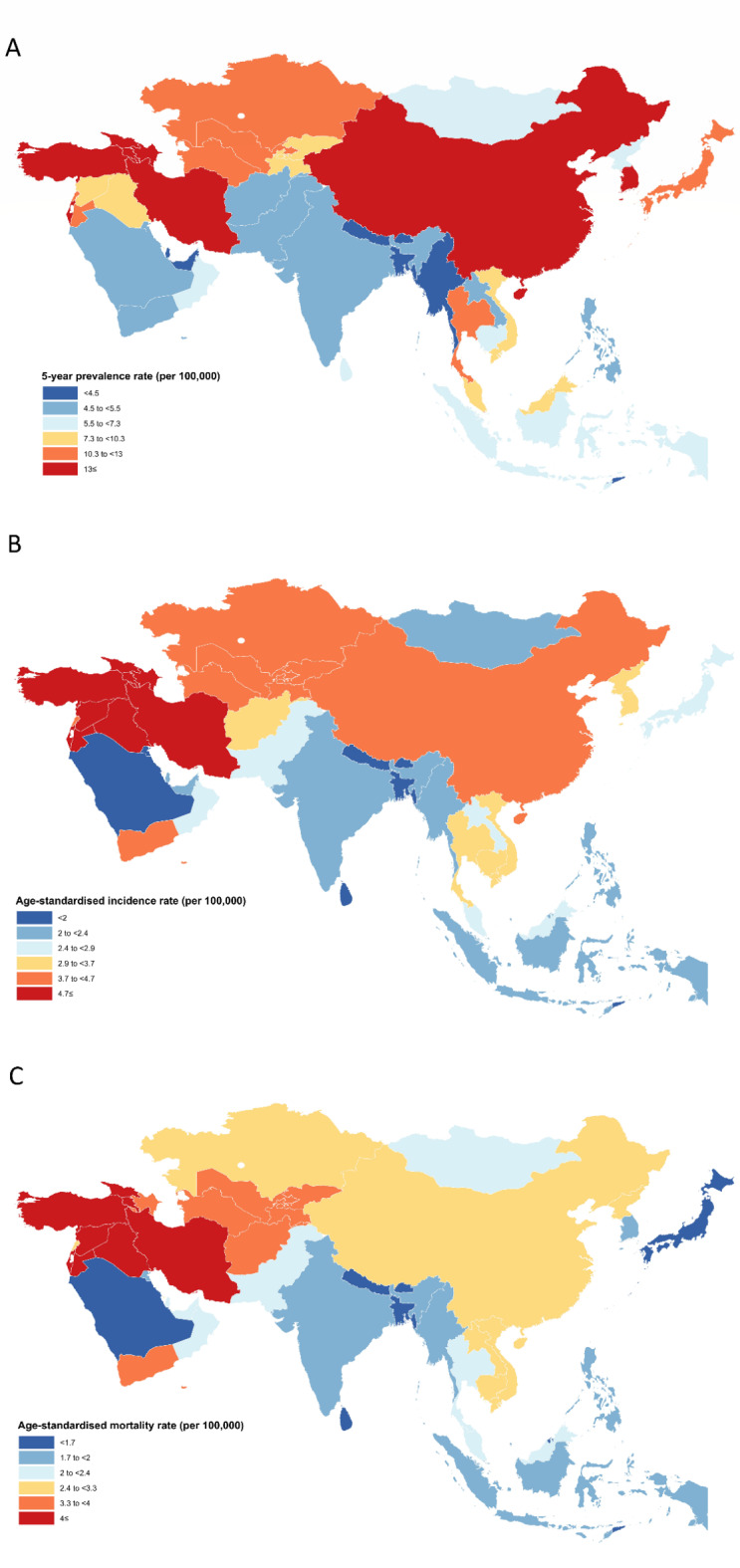
Distribution of (**A**) five-year prevalence rate, (**B**) age-standardized incidence and (**C**) age-standardized mortality rates per 100,000 of brain and central nervous system cancers among both sexes in Asia in 2020.

Among Asian countries, Armenia and Bangladesh, with estimated 5-year prevalence rates of 25.92 and 1.80, had the highest and lowest values, respectively (Fig. 1A). Additionally, Armenia had the highest ASIR per 100,000 (7.40), followed by Iran (7.20), Georgia (6.30), and Turkey (6.30) (Fig. 1B). The ASMR values were highest in Armenia, Iran, and Turkey at 6.20, 6.20, and 5.10 per 100,000, respectively (Fig. 1C). Country-specific metrics in Asia in both sexes are provided in Table 2.

**Table 2:** Five-year prevalence, incidence, and mortality metrics in 2020 of brain and central nervous system cancers for Asian countries in both sexes.

Location	Prevalence		Incidence					Mortality					MIR	Asia region
	5-year prevalent cases	5-year prevalence rate	Number	Uncertainty interval	Crude rate	ASIR	Cumulative risk (%)	Number	Uncertainty interval	Crude rate	ASMR	Cumulative risk (%)		
China	214,529	14.80	79,575	77240.90–81979.50	5.50	4.10	0.66	65,204	62152.20–68405.70	4.50	3.20	0.59	0.82	Eastern Asia
India	74,398	5.40	31,460	29527.70–33518.60	2.30	2.30	0.34	26,656	24914.90–28518.80	1.90	1.90	0.32	0.83	South-Central Asia
Turkey	17,034	20.20	6102	5253.50–7087.50	7.20	6.30	1.25	5070	4918.70–5225.90	6.00	5.10	1.13	0.83	Western Asia
Iran, Islamic Republic of	16,975	20.20	6180	5933.40–6436.90	7.40	7.20	1.76	5302	5128.50–5481.30	6.30	6.20	1.55	0.85	South-Central Asia
Japan	16,315	12.90	5517	4673.60–6512.60	4.40	2.70	0.39	3254	3082.10–3435.40	2.60	1.30	0.23	0.59	Eastern Asia
Indonesia	15,310	5.60	5964	4844.30–7342.50	2.20	2.10	0.30	5298	4288.60–6545.00	1.90	1.90	0.29	0.86	South-Eastern Asia
Pakistan	10,114	4.60	4770	3981.60–5714.50	2.20	2.60	0.34	3934	3260.60–4746.50	1.80	2.20	0.31	0.82	South-Central Asia
Viet Nam	8080	8.30	3120	2583.20–3768.30	3.20	2.90	0.37	2614	2123.60–3217.60	2.70	2.40	0.33	0.84	South-Eastern Asia
Thailand	7239	10.40	2734	2370.00–3153.90	3.90	2.90	0.51	2310	1943.30–2745.90	3.30	2.20	0.46	0.85	South-Eastern Asia
Korea, Republic of	6743	13.20	2153	1993.20–2325.70	4.20	3.00	0.46	1499	1388.80–1617.90	2.90	1.70	0.37	0.69	Eastern Asia
Philippines	5311	4.80	2037	1698.40–2443.10	1.90	2.00	0.37	1752	1403.00–2187.90	1.60	1.80	0.36	0.84	South-Eastern Asia
Iraq	4094	10.20	1600	1512.20–1692.90	4.00	5.50	0.90	1366	1269.60–1469.8	3.40	5.00	0.86	0.85	Western Asia
Uzbekistan	3725	11.10	1387	1258.80–1528.30	4.10	4.20	0.53	1190	1112.00–1273.50	3.60	3.70	0.48	0.88	South-Central Asia
Bangladesh	2898	1.80	1284	899.30–1833.30	0.78	0.85	0.11	1144	821.30–1593.40	0.69	0.76	0.11	0.88	South-Central Asia
Malaysia	2388	7.40	800	717.30–892.30	2.50	2.50	0.31	694	620.00–776.90	2.10	2.00	0.30	0.84	South-Eastern Asia
Kazakhstan	2243	11.90	746	684.30–813.30	4.00	3.70	0.47	629	572.40–691.10	3.30	3.10	0.42	0.83	South-Central Asia
Myanmar	2238	4.10	1057	354.40–3152.90	1.90	2.00	0.51	912	362.70–2292.90	1.70	1.80	0.50	0.89	South-Eastern Asia
Afghanistan	1935	5.00	1015	710.90–1449.20	2.60	3.60	0.47	897	644.00–1249.40	2.30	3.50	0.47	0.88	South-Central Asia
Israel	1912	22.10	619	546.40–701.20	7.20	5.80	1.03	493	420.00–578.60	5.70	4.30	0.92	0.79	Western Asia
Saudi Arabia	1876	5.40	598	479.30–746.10	1.70	1.90	0.28	486	358.10–659.50	1.40	1.50	0.28	0.83	Western Asia
Korea, Democratic Republic of	1807	7.00	1010	734.80–1388.20	3.90	3.10	0.52	817	548.00–1218.00	3.20	2.50	0.45	0.82	Eastern Asia
Azerbaijan	1487	14.70	542	470.50–624.40	5.30	4.70	0.74	460	388.40–544.80	4.50	3.90	0.69	0.85	Western Asia
Syrian Arab Republic	1429	8.20	710	204.20–2468.70	4.10	4.70	0.70	628	188.70–2089.70	3.60	4.20	0.67	0.88	Western Asia
Yemen	1386	4.60	803	499.90–1289.90	2.70	3.80	0.83	689	405.20–1171.60	2.30	3.50	0.83	0.85	Western Asia
Sri Lanka	1269	5.90	435	385.70–490.60	2.00	1.80	0.19	369	317.30–429.20	1.70	1.50	0.17	0.85	South-Central Asia
Cambodia	1122	6.70	514	172.30–1533.20	3.10	3.50	0.66	446	177.40–1121.30	2.70	3.10	0.64	0.87	South-Eastern Asia
Jordan	1103	10.80	415	337.30–510.60	4.10	5.00	0.76	336	255.10–442.50	3.30	4.20	0.72	0.81	Western Asia
Georgia	973	24.40	358	329.60–388.80	9.00	6.30	0.97	304	254.00–363.80	7.60	5.00	0.85	0.85	Western Asia
Armenia	768	25.90	291	267.90–316.10	9.80	7.40	1.19	252	205.50–309.00	8.50	6.20	1.05	0.87	Western Asia
Tajikistan	711	7.50	288	243.10–341.10	3.00	3.70	0.56	253	219.30–291.80	2.70	3.40	0.54	0.90	South-Central Asia
Lebanon	706	10.30	266	217.80–324.80	3.90	3.80	0.50	221	167.70–291.20	3.20	3.10	0.45	0.82	Western Asia
Singapore	704	12.00	218	163.50–290.60	3.70	2.80	0.45	164	132.30–203.30	2.80	1.90	0.32	0.76	South-Eastern Asia
Turkmenistan	626	10.40	231	190.10–280.70	3.80	3.90	0.42	211	178.10–250.00	3.50	3.60	0.40	0.92	South-Central Asia
Kyrgyzstan	613	9.40	244	199.90–297.90	3.70	4.10	0.57	210	177.80–248.10	3.20	3.60	0.53	0.86	South-Central Asia
Nepal	571	2.00	262	196.70–349.00	0.90	1.00	0.10	229	164.40–319.00	0.79	0.88	0.09	0.88	South-Central Asia
Lao People’s Democratic Republic	396	5.40	173	58.00–516.00	2.40	2.80	0.47	148	58.90–372.10	2.00	2.40	0.45	0.83	South-Eastern Asia
United Arab Emirates	383	3.90	119	84.60–167.40	1.20	2.10	0.49	98	62.90–152.60	0.99	2.20	0.74	0.83	Western Asia
Gaza Strip and West Bank	372	7.30	146	42.00–507.70	2.90	3.70	0.48	122	36.70–406.00	2.40	3.40	0.48	0.83	Western Asia
Oman	350	6.90	113	76.40–167.10	2.20	2.60	0.29	93	57.30–150.80	1.80	2.20	0.31	0.82	Western Asia
Kuwait	195	4.60	68	50.50–91.60	1.60	1.90	0.32	57	38.50–84.40	1.30	1.70	0.40	0.81	Western Asia
Mongolia	182	5.60	67	50.80–88.40	2.00	2.30	0.40	59	42.20–82.50	1.80	2.10	0.34	0.90	Eastern Asia
Bahrain	125	7.30	40	26.00–61.60	2.40	3.20	0.45	32	18.40–55.50	1.90	2.80	0.73	0.79	Western Asia
Qatar	122	4.20	39	13.90–109.40	1.40	2.10	0.29	31	9.30–103.20	1.10	2.30	0.66	0.79	Western Asia
Brunei Darussalam	33	7.50	11	5.00–24.10	2.50	2.10	0.23	7	2.70–18.20	1.60	1.50	0.18	0.64	South-Eastern Asia
Timor-Leste	31	2.40	15	5.00–44.70	1.10	1.40	0.33	16	6.40–40.20	1.20	1.60	0.36	1.09	South-Eastern Asia
Bhutan	23	3.00	12	9.70–14.80	1.60	1.60	0.47	11	8.90–13.70	1.40	1.50	0.46	0.88	South-Central Asia
Maldives	22	4.10	8	5.60–11.40	1.50	1.50	0.15	8	6.60–9.60	1.50	2.10	0.30	1.00	South-Central Asia

*ASIR* age-standardized incidence rate, *ASMR*: age-standardized mortality rate, *MIR* mortality-to-incidence ratio. Rates are presented per 100,000 population.

###  Age patterns

**Fig. 2:**
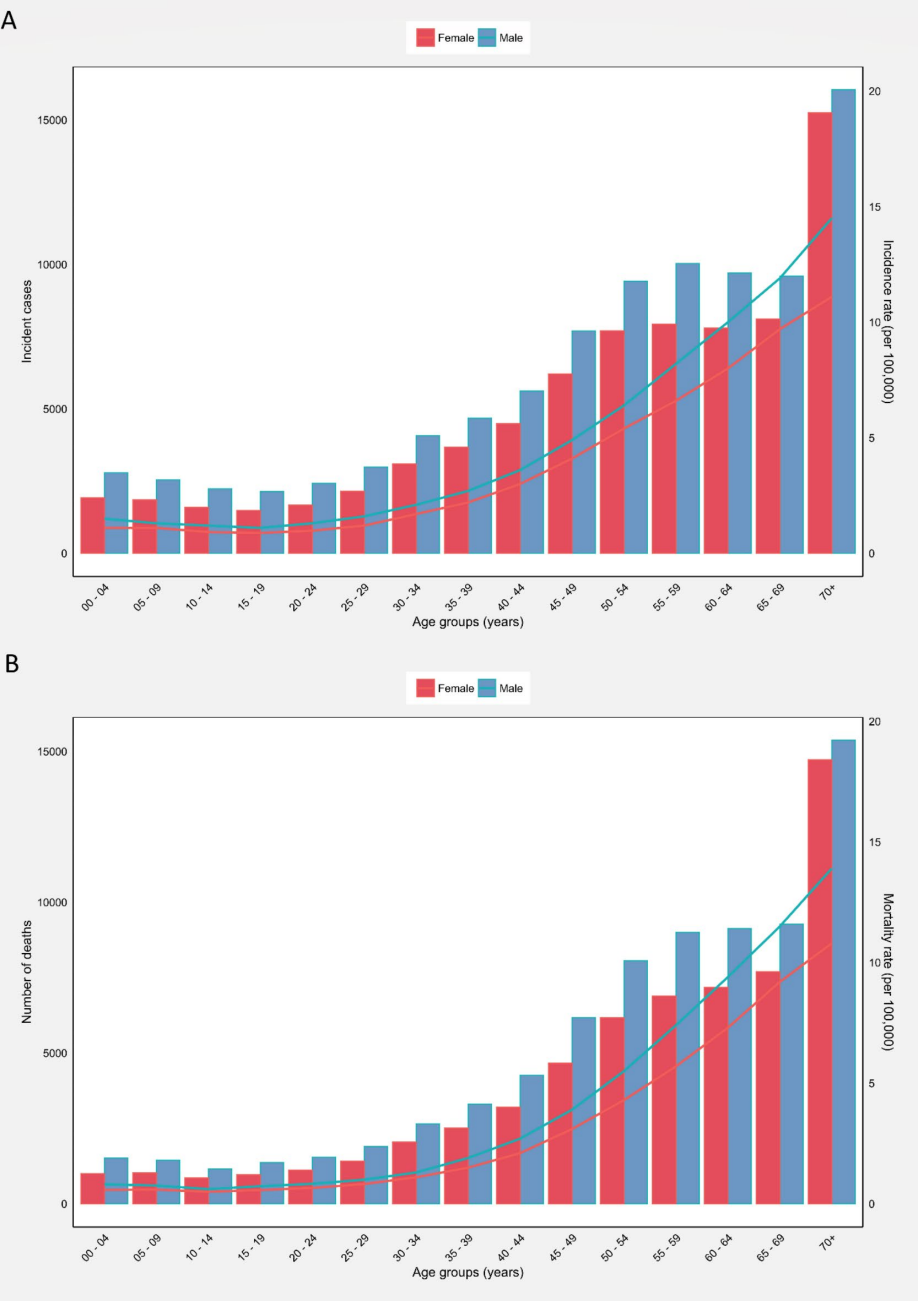
(**A**) Incident numbers and incidence rate, and (**B**) mortality numbers and mortality rate of brain and central nervous system cancers among males and females in each age group in Asia in 2020. The column bars represent the numbers of incident cases and deaths related to the left y-axis. The lines represent the incidence and mortality rates related to the right y-axis.

The 70 + age group accounted for the highest crude incidence rate (12.70 per 100,000) and crude mortality rate (12.20 per 100,000) in both sexes in Asia (Table 3). In terms of MIR, this age group also presented the highest MIR, 0.96. Moreover, people within the age range of 10–19 years presented the lowest crude incidence rate (1.00 per 100,000) and crude mortality rate (0.60 per 100,000). The lowest MIR belonged to the 0–9 age group (0.53). There were minimal variations in the incidence (Fig. 2A) and mortality rates (Fig. 2B) of brain and CNS cancers until the age of 40–49, after which there was a substantial increase in both incidence and mortality rates. The number of incident cases decreased up to the 15–19 year age group, then increased to the 55–59 year age group, and peaked in the 70 + year age group (Fig. 2A). Deaths generally exhibited an increasing trend with advancing age (Fig. 2B).

**Table 3:** Incidence, mortality, and mortality-to-incidence ratio metrics of brain and central nervous system cancers in Asia in 2020 for different age groups among both sexes, males, and females.

Age group	Incidence			Mortality			MIR
	Number	Crude rate	Cumulative risk (%)	Number	Crude rate	Cumulative risk (%)	
Both sexes							
0 to 9	9121	1.30	0.01	4991	0.69	0.01	0.53
10 to 19	7451	1.00	0.01	4341	0.60	0.01	0.60
20 to 29	9228	1.30	0.01	5982	0.83	0.01	0.64
30 to 39	15,524	2.20	0.02	10,523	1.50	0.01	0.68
40 to 49	24,010	3.90	0.04	18,320	3.00	0.03	0.77
50 to 59	35,082	6.60	0.07	30,116	5.70	0.06	0.86
60 to 69	35,210	9.80	0.10	33,279	9.30	0.09	0.95
70+	31,299	12.70	0.26	30,100	12.20	0.25	0.96
Males							
0 to 9	5340	1.40	0.01	2966	0.78	0.01	0.56
10 to 19	4374	1.20	0.01	2518	0.67	0.01	0.56
20 to 29	5411	1.40	0.01	3447	0.92	0.01	0.66
30 to 39	8755	2.40	0.02	5960	1.60	0.02	0.67
40 to 49	13,313	4.20	0.04	10,446	3.30	0.03	0.79
50 to 59	19,451	7.20	0.07	17,058	6.40	0.06	0.89
60 to 69	19,299	10.90	0.11	18,400	10.40	0.10	0.95
70+	16,049	14.50	0.31	15,374	13.90	0.29	0.96
Females							
0 to 9	3781	1.10	0.01	2025	0.58	0.01	0.53
10 to 19	3077	0.90	0.01	1823	0.53	0.01	0.59
20 to 29	3817	1.10	0.01	2535	0.74	0.01	0.67
30 to 39	6769	1.90	0.02	4563	1.30	0.01	0.68
40 to 49	10,697	3.50	0.04	7874	2.60	0.03	0.74
50 to 59	15,631	5.90	0.06	13,058	5.00	0.05	0.85
60 to 69	15,911	8.80	0.09	14,873	8.20	0.08	0.93
70+	15,250	11.10	0.23	14,726	10.80	0.22	0.97

*MIR* mortality-to-incidence ratio. Rates are presented per 100,000 population.

### Sex patterns

#### Men

In 2020, the estimated 5-year prevalence rate was 9.40 per 100,000 men in Asia. Our study also highlighted a total of 91,992 new male cases were estimated of brain and CNS cancers (95% UI: 86,579.5–97,742.8), representing a crude rate of 3.90 per 100,000, an ASIR of 3.50 per 100,000, and a cumulative risk of 0.59%. With respect to the mortality of Asian men, brain and CNS cancers resulted in 76,169 estimated deaths in 2020 (95% UI: 71,192.7–81,493.1). The crude mortality rate was estimated 3.20 per 100,000, with an ASMR of 2.90 per 100,000 and a cumulative risk of 0.53%. We measured the MIR of brain and CNS cancers as 0.82 in men in Asia (Table 1). Similar to the total population, the 70 + year age group presented the highest crude rates of incidence (14.50) and mortality (13.90) in men (Table 3).

**Fig. 3:**
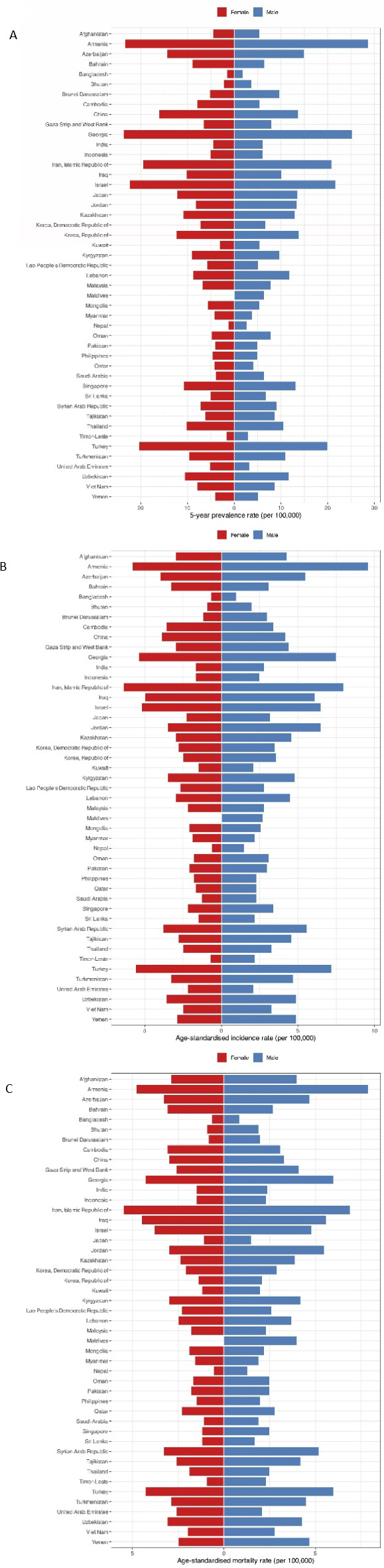
(**A**) Five-year prevalence rate, (**B**) age-standardized incidence and (**C**) age-standardized mortality rates per 100,000 of brain and central nervous system cancers in Asia in 2020, by country and sex.

Among Asian countries, the highest 5-year prevalence rate in men was 28.70 per 100,000, as estimated in Armenia (Fig. 3A, Fig. S1, and Supplementary File 1). Armenia (9.60), Iran (8.00), Georgia (7.50), and Turkey (7.20) presented the highest estimated ASIRs per 100,000 men (Fig. 3B, Fig. S2, and Supplementary File 1). The countries with the largest ASMRs in men also included Armenia (7.90), Iran (6.90), Georgia (6.00), and Turkey (6.00) per 100,000 (Fig. 3C, Fig. S3, and Supplementary File 1).

#### Women

Among women, the estimated 5-year prevalence rate of these cancers was 9.40 per 100,000. In this regard, 74,933 new cases were estimated of brain and CNS cancers (95% UI: 70,236.4–79,943.6). The crude incidence rate among women was 3.30 per 100,000, the ASIR was 2.80 per 100,000, and the cumulative risk was 0.46%. Brain and CNS cancers also led to 61,477 deaths among Asian women in 2020 (95% UI: 57,294.0–65,965.4), with a crude mortality rate of 2.70 per 100,000, an ASMR of 2.30 per 100,000, and a cumulative risk of 0.41%. The MIR of brain and CNS cancers was 0.82 in women, which was the same as that in men (Table 1). Moreover, compared with the total population and men, the 70 + age group presented the highest crude rates of incidence (11.10) and mortality (10.80) among the other age groups (Table 3).

The highest 5-year prevalence rate in women was 23.70 per 100,000, as estimated in Georgia (Fig. 3A, Fig. S4, and Supplementary File 1). The Asian countries with the highest values of ASIRs (per 100,000) were as follows: Iran (6.40), Armenia (5.80), Turkey (5.60), and Georgia (5.40) (Fig. 3B, Fig. S5, and Supplementary File 1). Furthermore, the countries with the highest ASMRs of women included Iran (5.50), Armenia (4.80), Iraq (4.50), Turkey, and Georgia (both 4.30) (Fig. 3C, Fig. S6, and Supplementary File 1).

###  Correlations with the HDI and CHE/GDP%

**Fig. 4:**
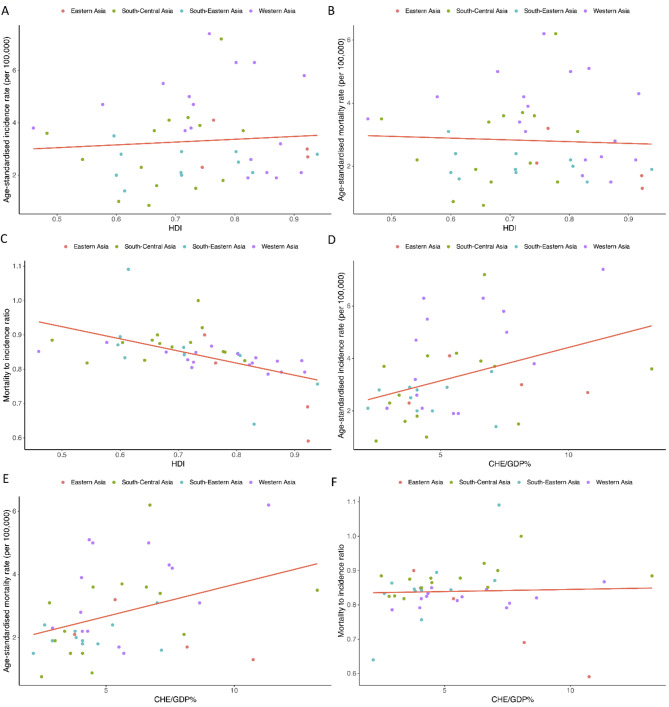
Association of human development index (HDI) with (**A**) age-standardized incidence rate (correlation coefficient: 0.079, p value: 0.603), (**B**) age-standardized mortality rate (correlation coefficient: -0.049, p value: 0.746), and (**C**) mortality-to-incidence ratio (correlation coefficient: − 0.538, p value < 0.001). Association of the current healthcare expenditure to gross domestic product (CHE/GDP%) with (**D**) age-standardized incidence rate (correlation coefficient: 0.388, p value: 0.010), (**E**) age-standardized mortality rate (correlation coefficient: 0.373, p value: 0.014), and (**F**) mortality-to-incidence ratio (correlation coefficient: 0.039, p value: 0.806) of brain and central nervous system cancers in Asia in 2020.

The HDI demonstrated a significant strong negative correlation with MIR (correlation coefficient: – 0.538, p value < 0.001; Fig. 4C). We also found moderately significant positive correlations between CHE/GDP% and ASIR (correlation coefficient: 0.388, p value: 0.010; Fig. 4D) and between CHE/GDP% and ASMR (correlation coefficient: 0.373, p value: 0.014; Fig. 4E). However, no significant correlations were found between HDI and ASIR (p value: 0.603, Fig. 4A) or ASMR (p value: 0.746, Fig. 4B) or between CHE/GDP% and MIR (p value: 0.806, Fig. 4F).

### Projections to 2040

**Fig. 5:**
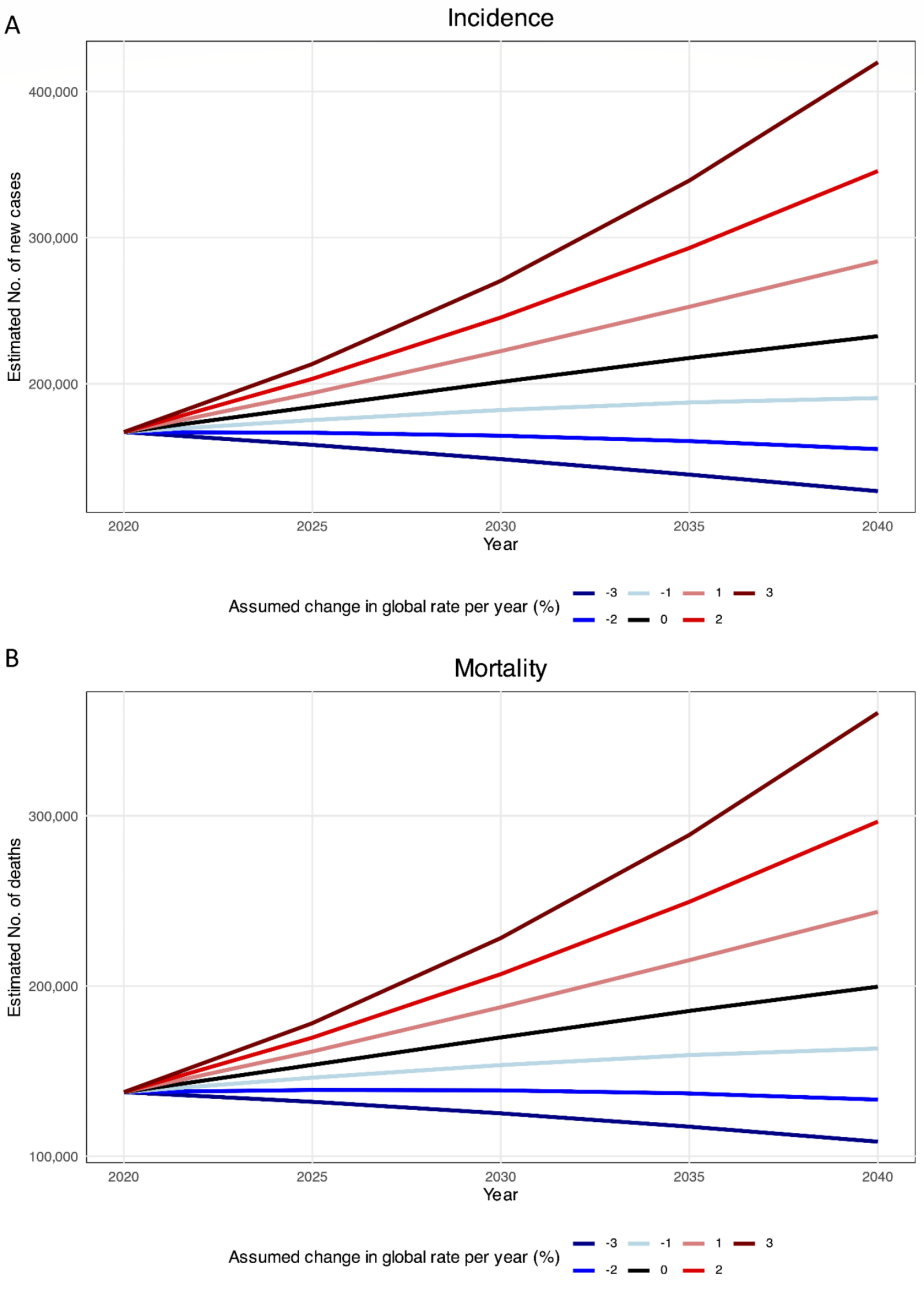
Estimated brain and central nervous system cancers (**A**) incidence and (**B**) mortality from 2020 to 2040. The baseline scenario (represented by the black line), posits that there are no alterations in incidence and mortality, meaning that any rise in numbers is solely attributed to changes in population size and composition. Due to the unlikelihood of stable incidence rates, alternative scenarios are provided.

We estimated that the number of newly diagnosed cases of brain and CNS cancers in Asia will increase by 39.30%, from 166,925 in 2020 to 232,000 cases in 2040 (Fig. 5A). Similarly, deaths caused by brain and CNS cancers are estimated to increase by 45.00%, from 137,646 in 2020 to 200,000 deaths in 2040 (Fig. 5B). There is a need for annual decreases of more than 1.60% in incidence and 1.80% in mortality in Asia to ensure that there would be fewer brain and CNS cancer cases in 2040 than in 2020.

## Discussion

In the present article, we provided an update on the epidemiology of brain and CNS cancer in Asia via the GLOBOCAN 2020 estimates. There were five main results in our study. First, we presented the prevalence, incidence, and mortality-related metrics of brain and CNS cancers in Asia, which are lower than those estimated worldwide. Second, we found that the countries with the highest ASIRs and ASMRs, namely, Armenia, Turkey, Iran, and Georgia, are all located in Western Asia and South Central Asia. Third, we revealed the age and sex patterns of these cancers, with the highest rates occurring in males and 70 + years. Fourth, we presented significant correlations of the assessed epidemiological metrics with the HDI and CHE/GDP%. Finally, we discussed the estimated projections of brain and CNS cancers that are expected to increase by approximately 40% until 2040.

In general, our estimates were consistent with trends in incidence and mortality reported in previous GBD studies, which evaluated the burden from 1990 to 2019^2,4^. Our ASIR estimates were slightly lower than those of GBD, possibly due to differences in data sources, estimation methods, and time periods^20^. Longitudinal analysis of the burden of brain and CNS cancers has similarly revealed increasing trends in the ASIR and decreasing trends in the ASMR across Asia and the globe^2,4^. Moreover, the GBD estimates from 1990 to 2019 revealed that West Asia always had the highest ASIRs, which aligns with our estimates in 2020. The ASIR was 6.01 among both sexes in Western Asia in 2019 in the GBD study, whereas it was 5.00 in Western Asia in 2020 in our study^4^. However, the highest levels of ASMRs were reported in Central Asia from 2005 until 2019^4^, which is not compatible with our findings, as we have shown that Western Asia still holds records of ASMRs in 2020. This might be explained by the fact that we used real data, whereas the GBD study uses estimation models. We also reported the values in 2020, while the GBD study used the data up to 2019.

As expected, the incidence and mortality of brain and CNS cancers exhibited an upward age trend, peaking after 40–49 years of age and maximizing in elderly individuals over 70 years of age. Similarly, the total glioma incidence increases with age, with a peak at 50–59 years of age^21^. Sex has also been shown to have a significant effect, with men being more likely to develop brain or CNS cancers in all age groups, possibly due to genetics and lifestyle factors^22^. This evidence highlights the role of age and sex as critical risk factors for brain and CNS cancers.

In this study, we revealed significant positive correlations between CHE/GDP% and both the ASIR and the ASMR alongside significant negative correlations between the HDI and MIR findings of brain and CNS cancers. As previously explained, CHE/GDP indicates the proportion of resources allocated to health care, and the HDI is a composite indicator of socioeconomic status^23^. Well-developed countries with higher HDIs possess more efficient healthcare systems that help with better surveillance and management of cancers^24^. Moreover, in these countries, better access to advanced medical treatments also affects the prognosis of patients^24^. One reason for the positive correlation between the ASMR and CHE/GDP% could be that, with better diagnosis, a larger proportion of overall mortality is attributed to brain and CNS cancers. A previous GLOBOCAN study has even shown that countries with different levels of HDI have distinct profiles of cancer incidence and mortality^25^. These findings suggest that countries with different socioeconomic statuses need tailored public health approaches to better manage brain and CNS cancers. Various diagnostic techniques and practices, unspecific genetic and environmental risk factors, and even races and ethnicities can account for such massive geographical and regional variation in the incidence and mortality of CNS cancer^26,27^. For example, countries with higher socioeconomic status have better technological capacity to correctly diagnose brain tumors, which may account for some of the variation. We used an approach that assumes an inverse linear relationship between the prevalence-to-incidence ratio and the HDI. This assumption is based on the understanding that regions with higher HDI typically have better healthcare systems, leading to earlier detection, more effective treatment, and thus higher survival rates, which result in a higher prevalence of survivors. This relationship has been observed in previous studies and provides a logical basis for our estimation method. However, we recognize that this assumption introduces potential limitations, as it may not account for all regional healthcare variations or other factors influencing cancer survival. Therefore, while this assumption is reasonable and supported by existing evidence, it requires careful consideration when interpreting the results.

We also forecasted an increasing trend in the incidence and mortality of brain and CNS cancers until 2040. Population aging is occurring more rapidly in Asia than in Western countries, and the number of Asian people older than 65 is estimated to increase to 857 million in 2050^28^. More efficient healthcare systems are needed to prevent, diagnose, and manage brain and CNS cancers. Apart from unchangeable risk factors (age, sex, and genetics), the only established modifiable factor increasing the risk of brain and CNS cancers is ionizing radiation^29^. More causal studies are needed to identify and assess numerous environmental risk factors to help better policies for cancer prevention. Enriched healthcare systems can also improve patient prognosis and reduce mortality^30^. However, one must note that enhanced hospital technologies and accessible professionals in developing countries might lead to higher incidence rates, which do not necessarily indicate a poorer health-related profile.

Our study has several limitations. First, there is a lack of epidemiological data on histopathological types of brain and CNS cancers. Different types of cancers exhibit considerable heterogeneity in terms of sex, age, race, and prognosis profiles. Second, low- and middle-income countries might have had less reliable data, leading to considerable uncertainties in estimates. Moreover, relevant data on brain and CNS cancer risk factors were not included in the database, and we could not explore the risk factors that contributed to the mortality of affected people. Additionally, the projections were calculated solely by assuming changes in the world population size and age structure, and we did not account for possible changes in age-specific incidence, globally or within countries. The projected incidence and mortality rates also do not take into account improvements in diagnostics or treatments. Third, one significant limitation in the epidemiology of CNS cancers is the challenge in identifying and collecting data on benign tumors. The ICD codes C70-C72 primarily cover malignant neoplasms, and while some cancer registries can identify and record benign tumors (D42-D44), others may either fail to collect this data or misclassify benign tumors as malignant. This inconsistency introduces bias, particularly in incidence and prevalence estimates, although it has a lesser impact on mortality rates. Also, the tumors of the pituitary or pineal glands were not included in this report, and can be considered in other studies. Fourth, in regions where national cancer registry data were incomplete or unavailable, we used the average incidence and mortality rates from adjacent countries to estimate figures for those countries. This approach was necessary to provide more comprehensive epidemiological data, but it may introduce some degree of bias or inaccuracy.

## Conclusions

Brain and CNS cancers remain major public health concerns in Asia. In our study, we presented the prevalence, incidence, mortality, and MIR according to sex, age, country, and Asian subregion. Moreover, we presented possible correlates with socioeconomics and estimated the epidemiological burden of these cancers in 2020. Overall, Western Asian, male, and elderly individuals had higher incidence rates and mortality rates. The findings of our study can help policymakers have a better perspective on the profile of brain and CNS cancers and enhance healthcare systems regarding the prevention, diagnosis, and management of these cancers. The burden attributable to each risk factor should be considered in future studies.

## Electronic supplementary material

Below is the link to the electronic supplementary material.

Supplementary Material 1

Supplementary Material 2

## Data Availability

The data used for these analyses are all publicly available at Global Cancer Observatory, the United Nations Development Programme (https://hdr.undp.org/data-center/human-development-index#/indicies/HDI), and the Global Health Observatory of the World Health Organization [https://www.who.int/data/gho/data/indicators/indicator-details/GHO/current-health-expenditure-(che)-as-percentage-of-gross-domestic-product-(gdp)-(-)].
