# Supplementary material for: Epidemiology and socioeconomic correlates of brain and central nervous system cancers in Asia in 2020 and their projection to 2040
Source: Sci Rep. 2024 Sep 20;14:21936. doi: 10.1038/s41598-024-73277-z (PMC11415511; doi:10.1038/s41598-024-73277-z)
Supplement: Supplementary file 1 — Supplementary Material 1 [file 41598_2024_73277_MOESM1_ESM.docx]

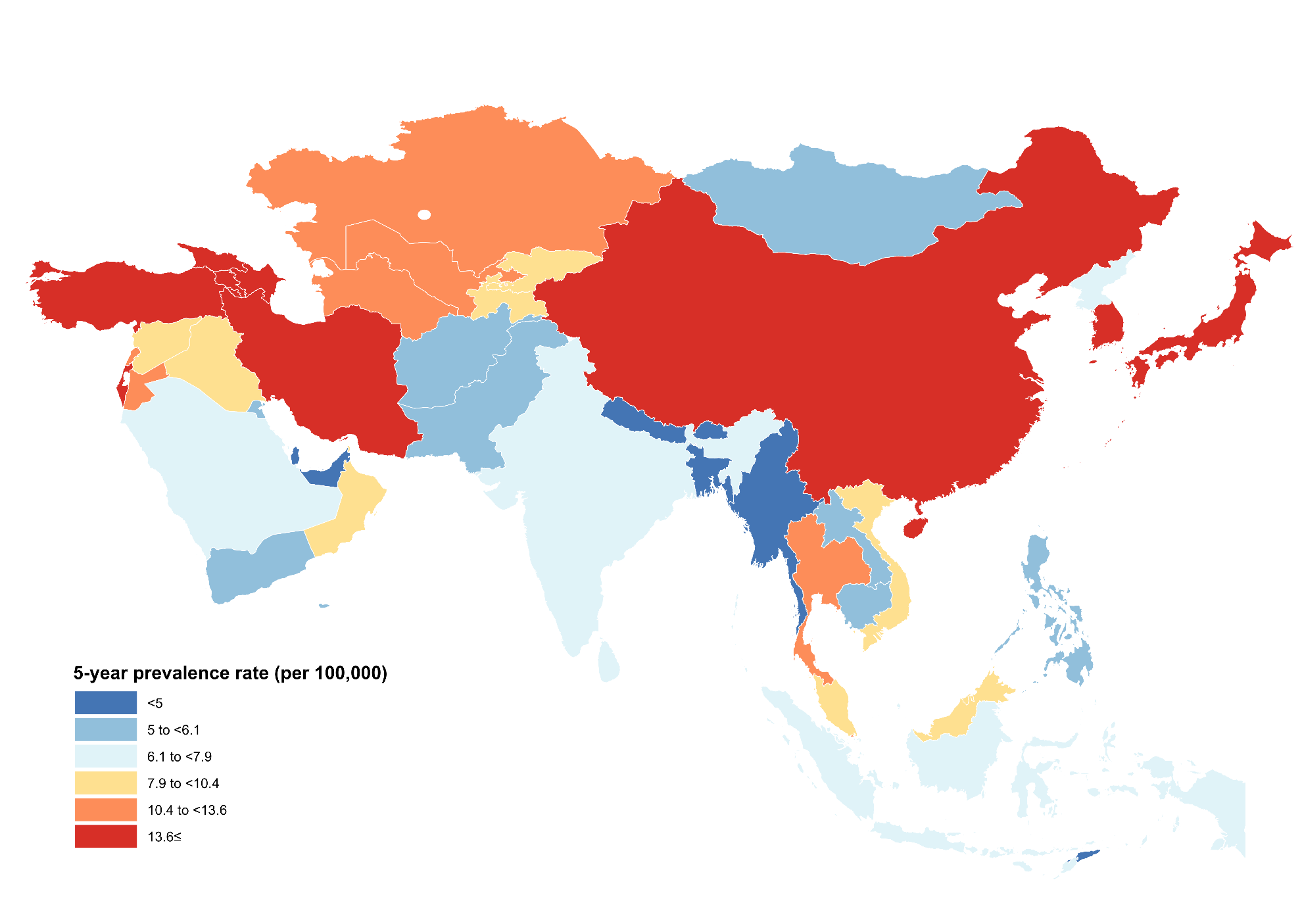


**Figure S1.** Distribution of five-year prevalence rate of male brain and central nervous system cancers in 2020 in Asia.


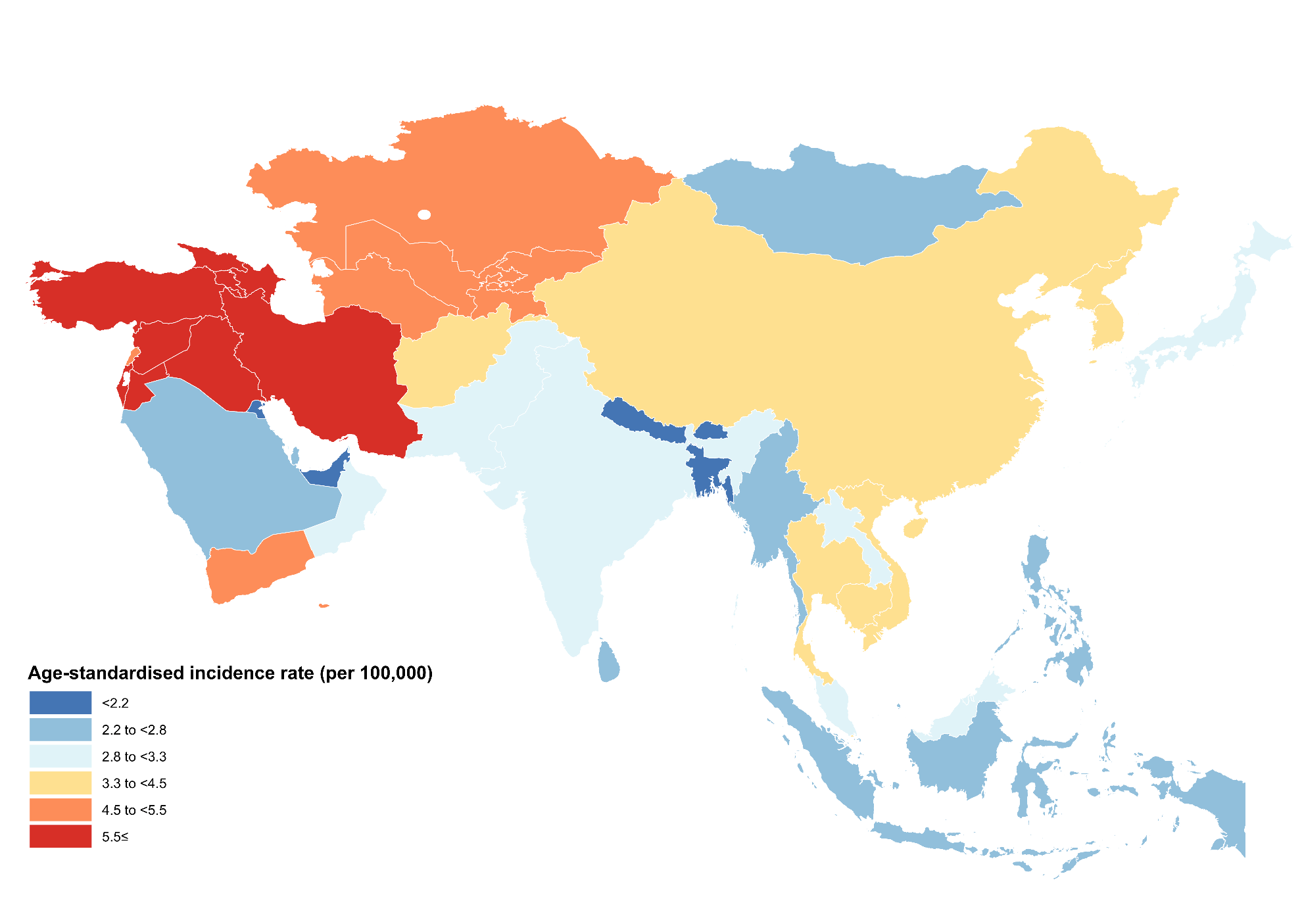


**Figure S2.** Distribution of age-standardized incidence rate of male brain and central nervous system cancers in 2020 in Asia.


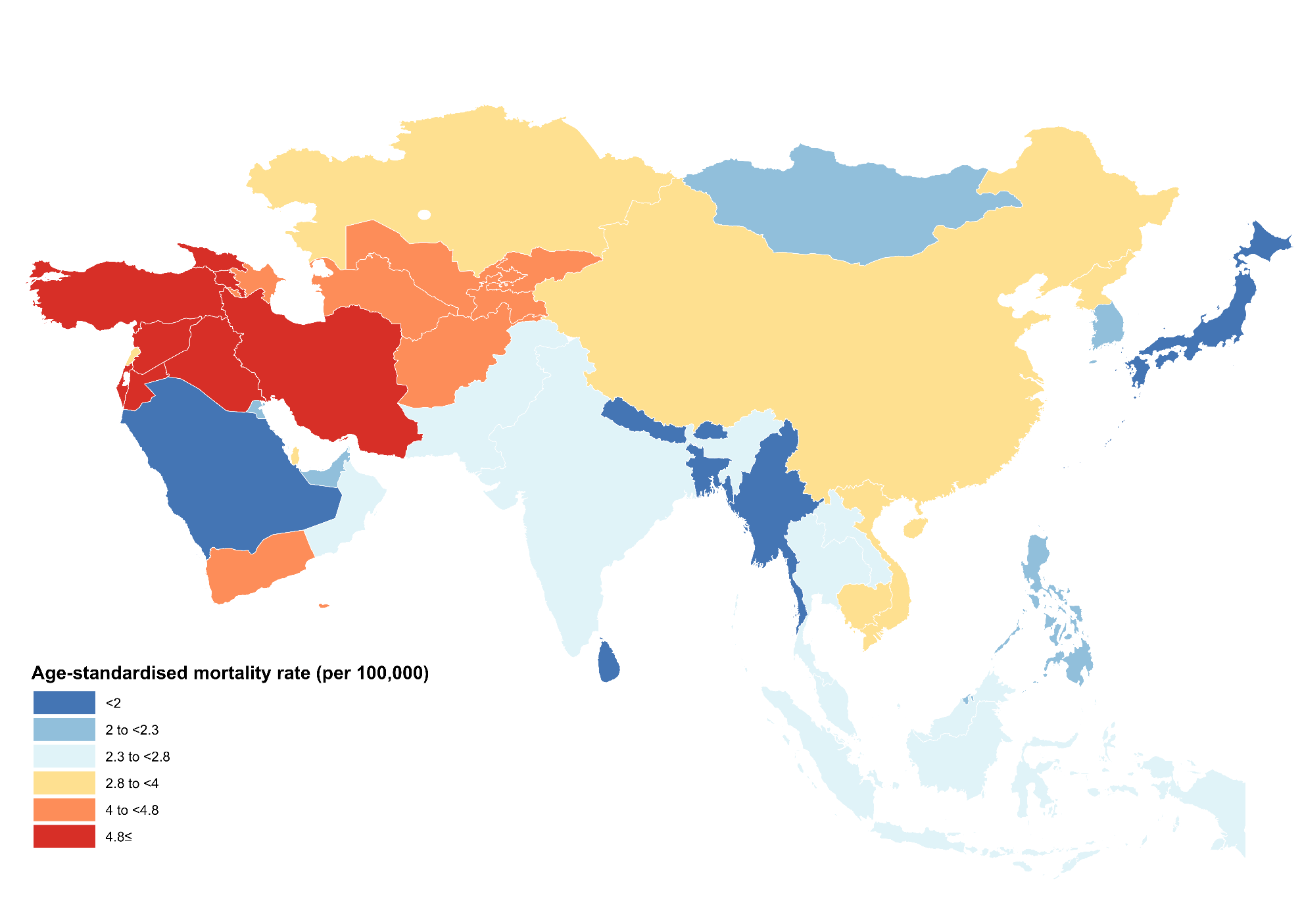


**Figure S3.** Distribution of age-standardized mortality rate of male brain and central nervous system cancers in 2020 in Asia.


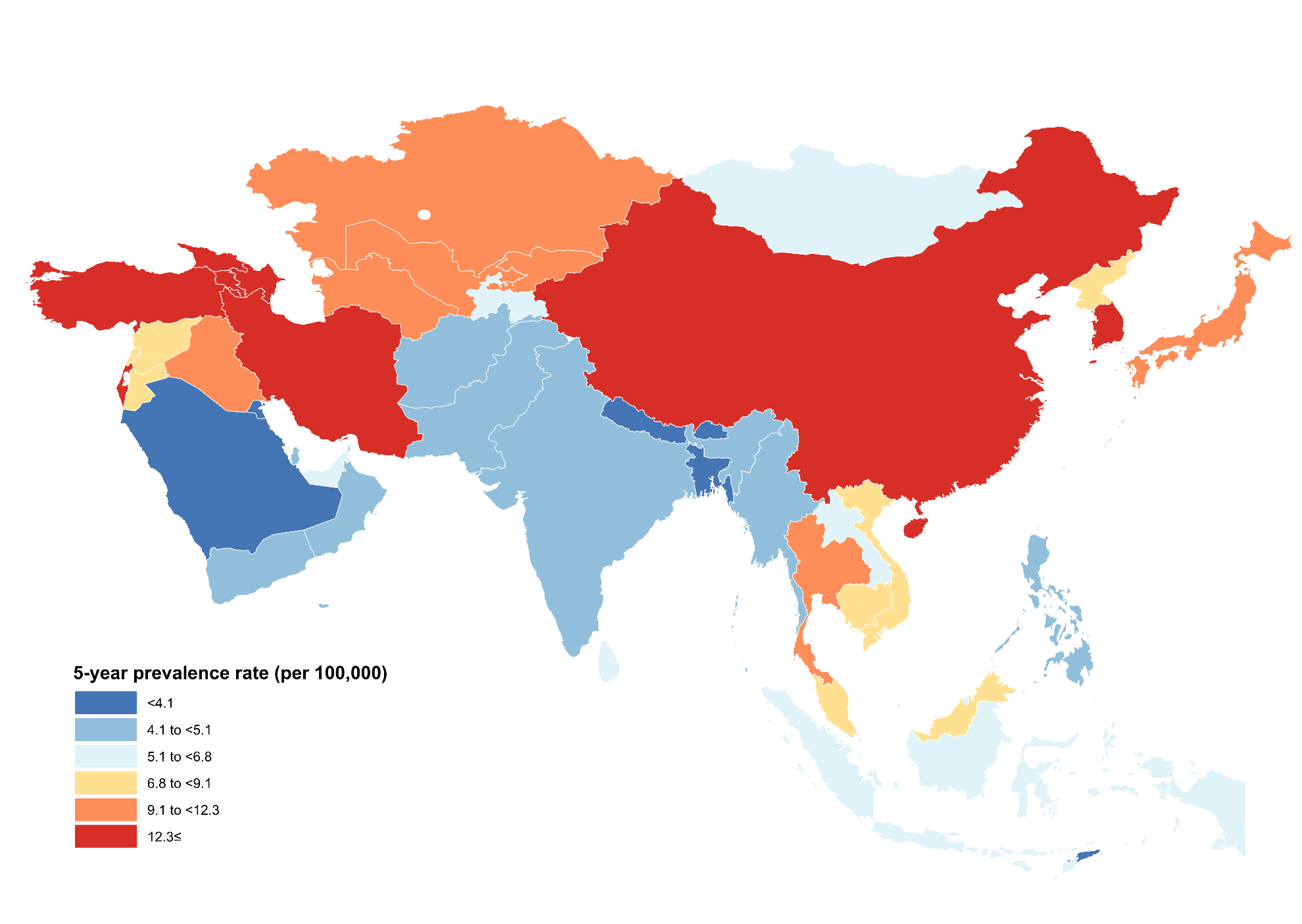


**Figure S4.** Distribution of five-year prevalence rate of female brain and central nervous system cancers in 2020 in Asia.


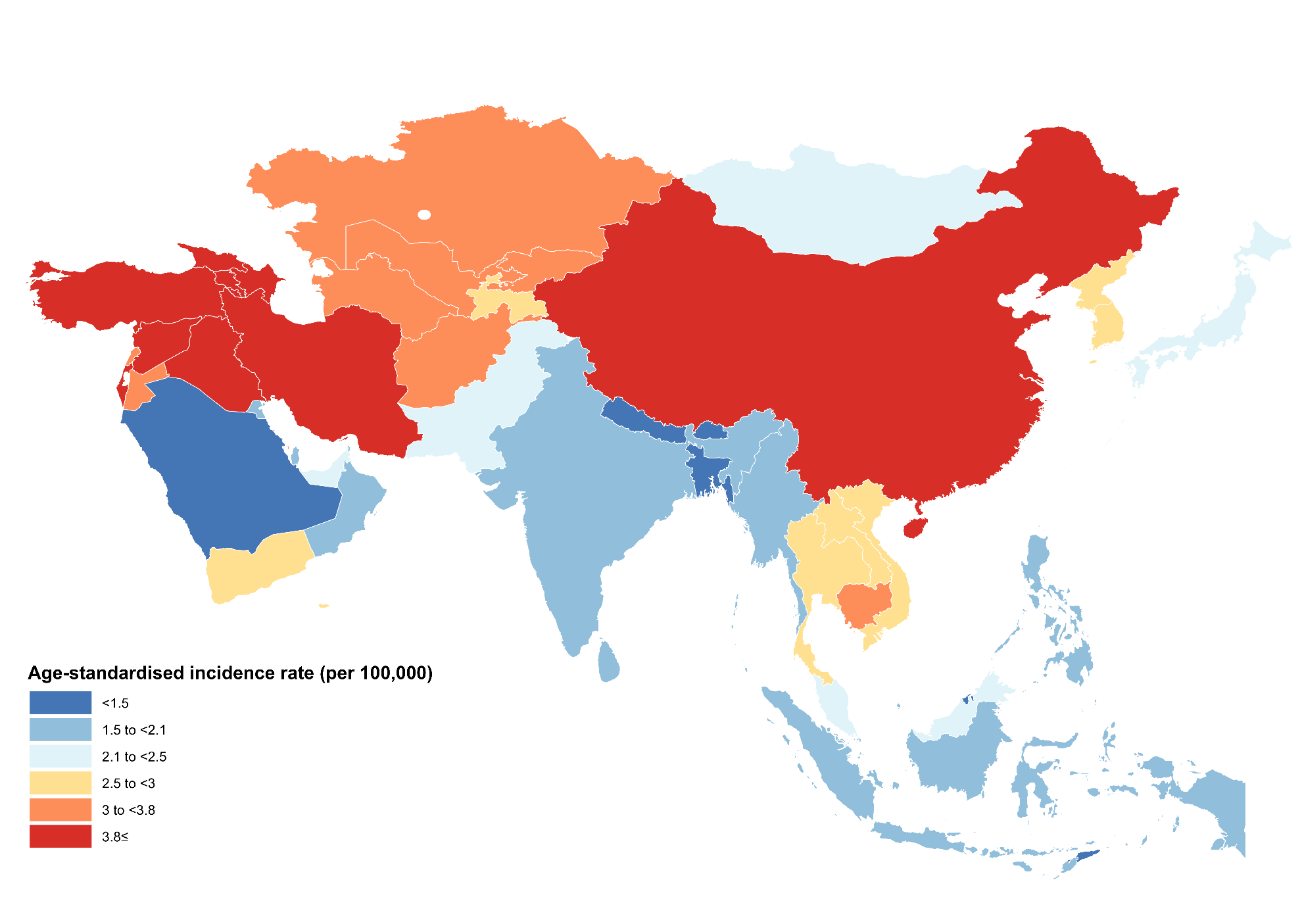


**Figure S5.** Distribution of age-standardized incidence rate of female brain and central nervous system cancers in 2020 in Asia.


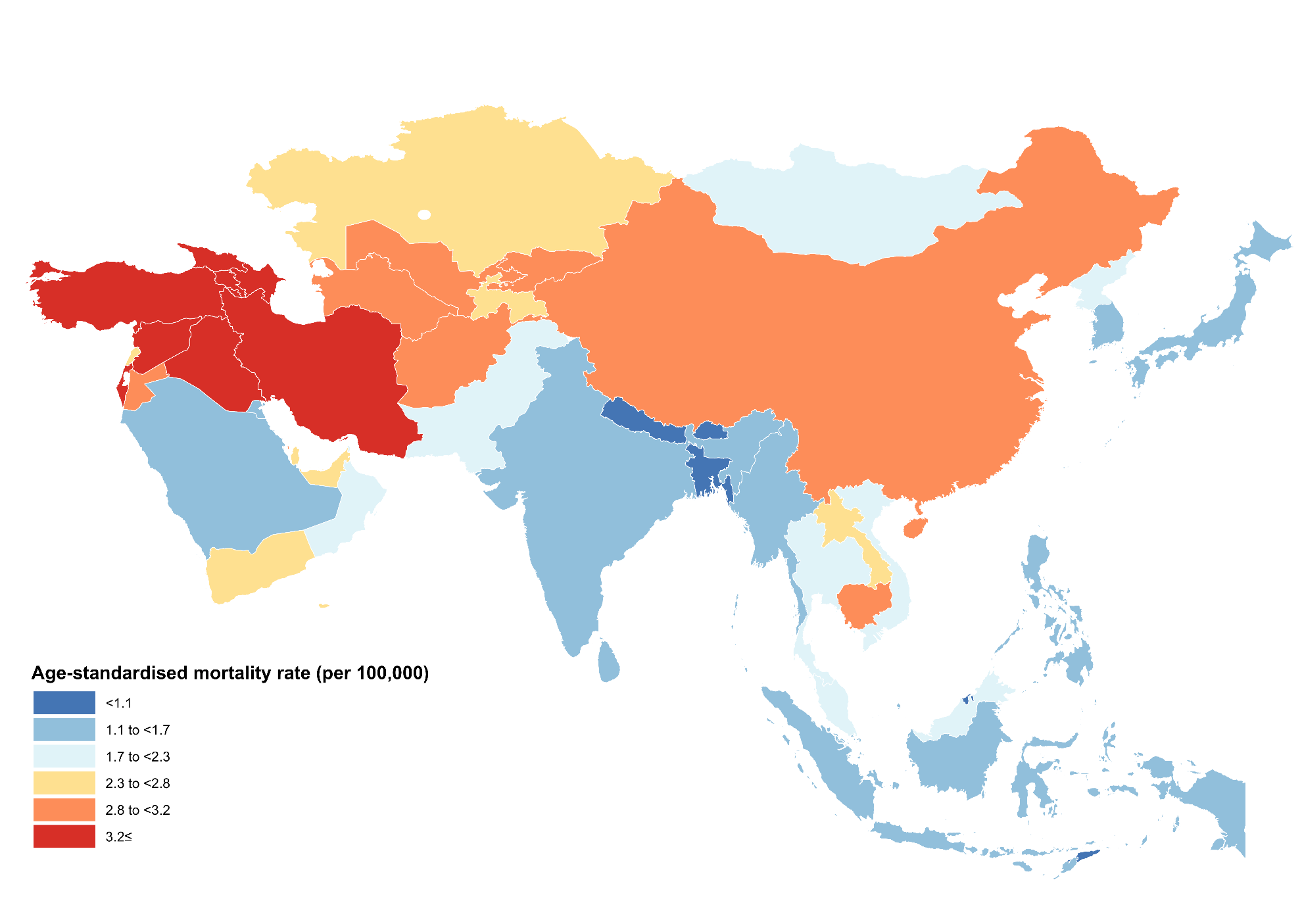


**Figure S6.** Distribution of age-standardized mortality rate of female brain and central nervous system cancers in 2020 in Asia.
